# Is Proton Therapy a “Pro” for Breast Cancer? A Comparison of Proton vs. Non-proton Radiotherapy Using the National Cancer Database

**DOI:** 10.3389/fonc.2018.00678

**Published:** 2019-01-14

**Authors:** Mudit Chowdhary, Anna Lee, Sarah Gao, Dian Wang, Parul N. Barry, Roberto Diaz, Neeti R. Bagadiya, Henry S. Park, James B. Yu, Lynn D. Wilson, Meena S. Moran, Susan A. Higgins, Christin A. Knowlton, Kirtesh R. Patel

**Affiliations:** ^1^Department of Radiation Oncology, Rush University Medical Center, Chicago, IL, United States; ^2^Department of Radiation Oncology, SUNY Downstate Medical Center, Brooklyn, NY, United States; ^3^Department of Therapeutic Radiology, Smilow Cancer Center, Yale University School of Medicine, New Haven, CT, United States; ^4^Department of Radiation Oncology, Moffitt Cancer Center, Tampa, FL, United States; ^5^Department of Radiology, Emory University School of Medicine, Atlanta, GA, United States

**Keywords:** proton, radiotherapy, breast cancer, patterns of care, overall survival

## Abstract

**Background:** Limited data exists demonstrating the clinical benefit of proton radiotherapy (PRT) in breast cancer. Using the National Cancer Database, we evaluated predictors associated with PRT use for patients with breast cancer. An exploratory analysis also investigates the impact of PRT on overall survival (OS).

**Methods:** Patients with non-metastatic breast cancer treated with adjuvant radiotherapy from 2004 to 2014 were identified. Patients were stratified based on receipt of PRT or non-PRT (i.e., photons ± electrons). A logistic regression model was used to determine predictors for PRT utilization. For OS, Multivariable analysis (MVA) was performed using Cox proportional hazard model.

**Results:** A total of 724,492 patients were identified: 871 received PRT and 723,621 received non-PRT. 58.3% of the PRT patients were group stage 0–1. Median follow-up time was 62.2 months. On multivariate logistic analysis, the following factors were found to be significant for receipt of PRT (all *p* < 0.05): academic facility (odds ratio [OR] = 2.50), South (OR = 2.01) and West location (OR = 12.43), left-sided (OR = 1.21), ER-positive (OR = 1.59), and mastectomy (OR = 1.47); pT2-T4 disease predicted for decrease use (OR = 0.79). PRT was not associated with OS on MVA for all patients: Hazard Ratio: 0.85, *p* = 0.168. PRT remained not significant on MVA after stratifying for subsets likely associated with higher heart radiation doses, including: left-sided (*p* = 0.140), inner-quadrant (*p* = 0.173), mastectomy (*p* = 0.095), node positivity (*p* = 0.680), N2-N3 disease (*p* = 0.880), and lymph node irradiation (LNI) (*p* = 0.767).

**Conclusions:** Receipt of PRT was associated with left-sided, ER+ tumors, mastectomy, South and West location, and academic facilities, but not higher group stages or LNI. PRT was not associated with OS, including in subsets likely at risk for higher heart doses. Further studies are required to determine non-OS benefits of PRT. In the interim, given the high cost of protons, only well-selected patients should receive PRT unless enrolled on a clinical trial.

## Introduction

Radiotherapy (RT) for breast cancer has been shown by the Early Breast Cancer Trialists' Collaborative Group meta-analyses to improve both progression free survival and breast cancer mortality (1). However, one of the competing risks for overall survival (OS) is radiation dose delivered to the heart: Darby et al. demonstrated a 7.4% increase in major coronary events with every 1 Gy increase in mean heart dose (2). To maximize the therapeutic risk/benefit ratio of RT for breast cancer, multiple irradiation techniques have been developed to decrease dose to the heart, including breath-hold technique, prone positioning, heart block, utilizing electron beam with photons, and intensity modulated RT (3). However, in certain patients—left sided, inner quadrant, node positive, undergoing mastectomy, and receiving internal mammary nodal irradiation—mean heart radiation doses still remain relatively high (4).

Proton radiotherapy (PRT) is a form of RT that has a lower exit dose due to a sharp dose gradient fall off, termed the Bragg peak (5). Multiple dosimetric and small clinical studies have suggested that protons lead to lower mean heart doses relative to non-PRT, including in high risk patients (6–12). Building on this potential, a prospective non-randomized study by MacDonald et al. demonstrated the feasibility of PRT in 12 breast cancer patients (13). At this time, however, PRT is not accessible to all patients. Moreover, randomized data demonstrating the benefit of PRT is not currently present.

The National Cancer Data Base (NCDB) is a large, prospectively acquired database that includes ~70% of newly diagnosed cancer patients treated at over 1,500 facilities in the United States (14–18). The NCDB records the RT modality, including PRT. Given the promise of protons, the aim of this study is to investigate utilization patterns of PRT for breast cancer. Furthermore, with evidence lacking on the survival benefit of proton therapy, an exploratory analysis comparing OS between PRT and non-proton RT, termed external beam radiotherapy (EBRT), for breast cancer patients was also performed.

## Materials and Methods

### Patient Selection

The NCDB 2015 Participant User File (PUF) for breast cancer was obtained for this analysis, which included patients diagnosed between 2004 and 2014. Review by the institutional review board was not required as this research study utilized the National Cancer Data Base (NCDB), which is a multi-institutional, de-identified cancer registry. Informed consent is also not applicable.

**Figure 1:**
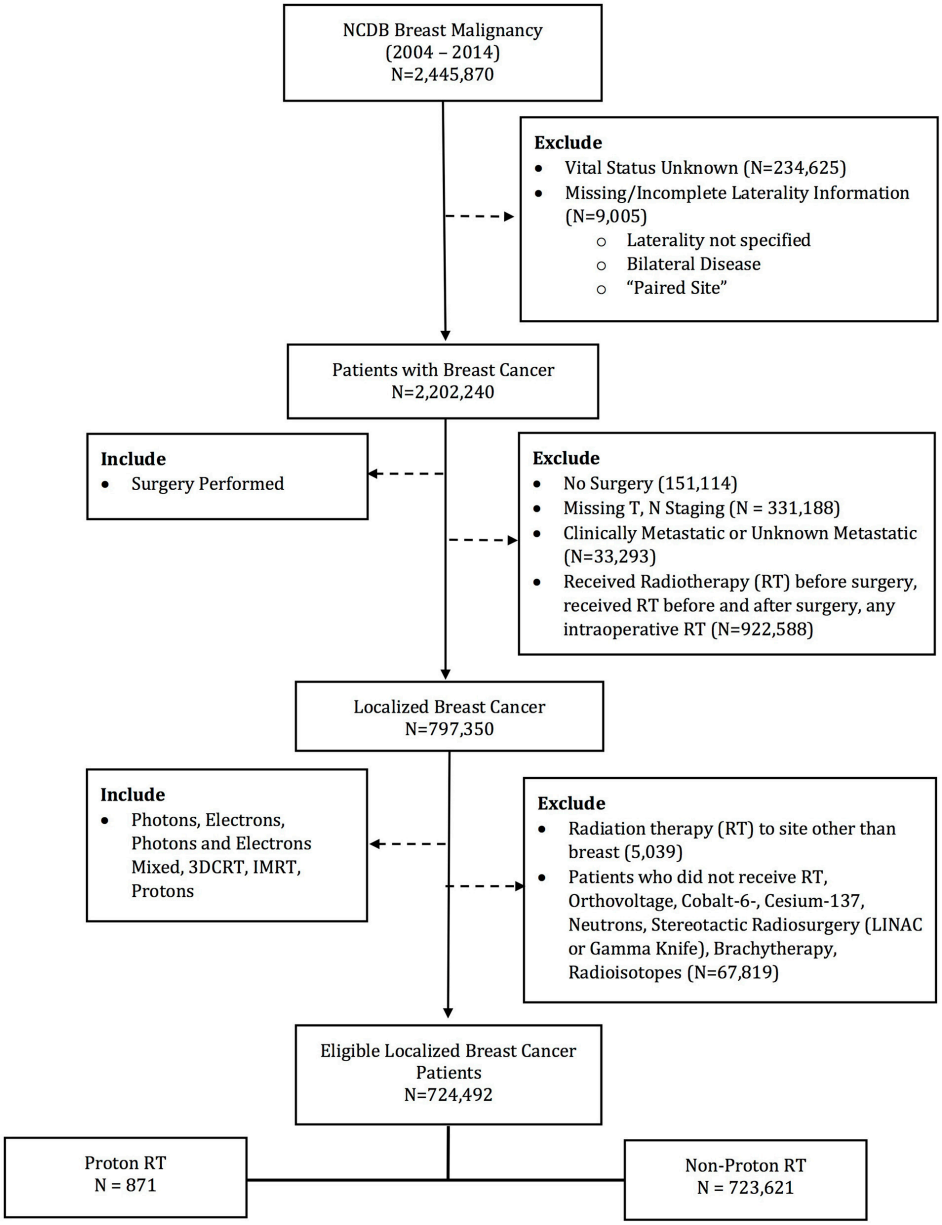
Consort Diagram for selection of patient cohort.

The database was queried for patients with stage 0–III breast patients undergoing surgery and post-operative radiotherapy. Patients receiving EBRT to the breast and regional lymph nodes were included. Exclusion criteria included patients with metastatic disease at diagnosis and without survival outcomes. Other exclusion criteria were not undergoing surgery or RT, receiving RT to a site other than breast, any RT prior to surgery, RT dose < 39 or >70 Gy, or non-EBRT modalities (i.e., brachytherapy, intraoperative radiotherapy, stereotactic radiosurgery, and radioisotopes). Patient demographics, socioeconomic status, disease characteristics, treatment details, and treatment outcomes were available for analysis. Patients were stratified into two groups: PRT and EBRT (Figure 1). The EBRT cohort included patients treated with photons alone or photons and electron boost.

### Patient Demographics

Patient's age at diagnosis, gender, race, type of health insurance, geographic location, education, median income quartile, treatment facility type were available for analysis. Charlson-Deyo Score was used as a surrogate marker for patient co-morbidities (19).

### Disease Characteristics

The following disease related variables were evaluated: diagnosis year, American Joint Cancer Committee (AJCC) pathological tumor and nodal stage, tumor laterality (left vs. right), quadrant location (inner quadrant vs. outer), and estrogen receptor (ER) status. Stratification criteria included nodal stage, laterality, and quadrant location. Patients were staged based on the AJCC staging edition in use during the year in which the case was diagnosed.

### Treatment Details

Patients were eligible if they underwent surgery and adjuvant RT. Type of surgery—breast conservation vs. mastectomy—was utilized as a stratification criterion.

Only patients receiving radiation to the breast or breast/chest wall ± lymph nodes were included and also used as stratification criteria. Receipt of hormonal therapy was also recorded, as was receipt and type (multi-agent vs. single-agent) of chemotherapy.

### Outcome

The primary outcome of this study was to analyze utilization patterns of PRT for breast cancer patients. An exploratory analysis compared OS between PRT and EBRT. OS was defined as time from diagnosis to time of death or last follow-up.

### Statistical Analysis

Clinical, pathological, treatment, and socioeconomic factors were compared between patients who received adjuvant PRT to those who received adjuvant EBRT using the Chi-square test or Mann-Whitney's test where appropriate. Univariate and multivariate logistic regression models were used to determine predictors for utilization of PRT.

Univariate associations between each variable and the two study cohorts (PRT and EBRT) were calculated using the χ^2^ test for categorical covariates and ANOVA for numerical covariates.

OS was estimated by the Kaplan Meier analysis and compared using Cox proportional hazard model and log-rank test. Univariate association between each variable and OS was performed. A multivariate Cox proportional hazard model for OS was fit using the backward selection method and a removal criterion of 0.20. Hazard ratios (HR) with associated 95% confidence interval (CI) were generated for each covariate and the outcome. All statistical analyses were conducted using SAS 9.4 (Cary, NC). All statistical analyses were two-sided, with *p*-values < 0.05 considered statistically significant.

## Results

### Baseline Characteristics

724,492 patients (720,147 [99.4%] females; 4,345 [0.6%] males) were identified in the NCDB Breast PUF 2015 file that met the specified inclusion criteria. 871 (0.12%) received PRT, while the remaining received EBRT. Median total radiation dose with PRT was 60.0 Gy, while median total radiation dose with EBRT was 60.4. The PRT cohort had multiple socioeconomic difference with the EBRT cohort, including a higher percentage of Caucasian race, private insurance, median income >$63,000, treatment at an academic facility, metropolitan residence and West location (all *p* < 0.05). However, patient age (59 vs. 60 years, *p* = 0.074) and education status (% with high school education: 60.9 vs. 63.4%, *p* = 0.137) were not statistically different. Among the PRT patients, 58.3% of were group stage 0–1 and 73.1% were pathologic T stage 0–1. Clinical and pathologic differences were also present between the two cohorts, with the PRT cohort having a higher percentage of group stage 0–1 patients, undergoing mastectomy, and left sided tumors. Rates of LNI were similar between the cohorts. Supplemental Table 1 demonstrates the comparison of the factors between cohorts. Median follow up for the entire cohort was 62.2 months. PRT patients had longer median follow up, 74.6 vs. 62.2 months, *p* < 0.001.

### Receipt of Proton Therapy

Table 1 describes factors that predict for receipt of PRT. Univariate logistic regression model demonstrated multiple socioeconomic factors associated with receipt of PRT, including Caucasian race, treatment at an academic facility, median income >63,000, South and West location, and metropolitan location. Clinical and pathological factors also predicted for receipt of PRT on univariate analysis, including year of diagnosis, lower T stage, lower group stage, mastectomy, ER positivity and receipt of endocrine therapy. MVA logistic regression model demonstrated academic facility [odds ratio (OR): 2.50, 95% confidence interval (CI): 2.15–2.90, *p* < 0.001], South (OR: 2.01; 95% CI: 1.50–2.70, *p* < 0.001) and West location (OR: 12.43, 95% CI: 9.62–16.05, *p* < 0.001) were predictive for receipt of PRT. Clinical and pathologic features predictive for PRT use included left-sided tumors (OR: 1.21; 95% CI: 1.05–1.39, *p* = 0.010), ER positive (OR: 1.59; 95% CI: 1.20-2.09, p = 0.001), receipt of endocrine therapy (OR: 0.70; 95% CI: 0.59–0.84, *p* < 0.001), and undergoing mastectomy (OR: 1.47; 95% CI: 1.20–1.81, *p* < 0.001). Higher T stage (pT2-T4 vs. pT0-pT1) predicted for decrease use of PRT (OR: 0.79; 95% CI: 0.66–0.96, *p* = 0.015); however, N stage and overall group stage did not predict for PRT usage. LNI also did not predict for PRT utilization.

**Table 1:** Univariate and multi-variate regression analysis to identify factors that predict for utilization of protons, relative to non-protons, in breast cancer patients.

	**Univariate**		**Multivariate**	
	**Odds ratio (95% CI)**	***P*-value**	**Odds ratio (95% CI)**	***P*-value**
**AGE**				
≤ 65	1		–	–
>65	0.92 (0.79–1.06)	0.236	–	–
**RACE**				
White	1		1	
Black	0.57 (0.44–0.75)	< 0.001	0.80 (0.58–1.09)	0.150
Other	1.73 (1.37–2.18)	< 0.001	0.79 (0.61–1.02)	0.071
**INSURANCE STATUS**				
None	1		–	–
Private	1.64 (0.90–2.98)	0.106	–	–
Medicaid	1.39 (0.72–2.67)	0.326	–	–
Medicare	1.35 (0.74–2.48)	0.325	–	–
Other govt/unknown	1.12 (0.52–2.44)	0.772	–	–
**CHARLSON-DEYO COMORBIDITY SCORE**				
0	1		1	
1	0.58 (0.45–0.76)	< 0.001	0.74 (0.57–0.97)	0.028
≥2	1.01 (0.65–1.57)	0.981	1.20 (0.75–1.92)	0.458
**FACILITY**				
Non-academic	1		1	
Academic	1.74 (1.52–2.00)	< 0.001	2.50 (2.15–2.90)	< 0.001
**MEDIAN HOUSEHOLD INCOME**				
< $38,000	1		1	
$38,000–$47,999	1.06 (0.81–1.39)	0.691	0.85 (0.64–1.14)	0.286
$48,000–$62,999	0.97 (0.74–1.26)	0.803	0.66 (0.50–0.88)	0.005
≥$63,000	2.10 (1.67–2.65)	< 0.001	1.28 (0.99–1.66)	0.061
**LOCATION**				
Northeast	1		1	
Midwest	1.06 (0.77–1.44)	0.733	1.31 (0.95–1.80)	0.097
South	1.50 (1.14–1.99)	0.004	2.01 (1.50–2.70)	< 0.001
West	9.79 (7.67–12.49)	< 0.001	12.43 (9.62–16.05)	< 0.001
**RESIDENCE**				
Metropolitan	1		1	
Urban	0.35 (0.26–0.49)	< 0.001	0.55 (0.39–0.78)	0.001
Rural	0.52 (0.25–1.10)	0.089	0.98 (0.46–2.08)	0.955
**PERCENTAGE WITH NO HIGH sCHOOL EDUCATION**				
> = 13%	1		–	–
< 13%	0.90 (0.79–1.03)	0.132	–	–
**LATERALITY**				
Right	1		1	
Left	1.15 (1.01–1.32)	0.035	1.21 (1.05–1.39)	0.010
**pT-STAGE**				
pT0-pT1	1		1	
pT2-pT4	0.88 (0.76–1.02)	0.092	0.79 (0.66–0.96)	0.015
**pN-STAGE**				
pN0	1		–	–
pN1	0.88 (0.74–1.05)	0.152	–	–
pN2-pN3	1.11 (0.90–1.36)	0.326	–	–
**OVERALL STAGE**				
0–I	1		–	–
II–III	0.92 (0.80–1.06)	0.228	–	–
**RECEPTOR STATUS**				
ER–	1		1	
ER+	1.36 (1.10–1.69)	0.005	1.59 (1.20–2.09)	0.001
Borderline/Unknown	3.46 (2.70–4.42)	< 0.001	2.97 (2.18–4.06)	< 0.001
**CHEMOTHERAPY**				
No	1		1	
Yes	0.89 (0.78–1.02)	0.093	0.89 (0.75–1.05)	0.174
**ENDOCRINE THERAPY**				
No	1		1	
Yes	0.75 (0.65–0.87)	< 0.001	0.70 (0.59–0.84)	< 0.001
**SURGERY**				
Breast conservation	1		1	
Mastectomy	1.21 (1.04–1.42)	0.016	1.47 (1.20–1.81)	< 0.001
**LYMPH NODE IRRADIATION**				
No	1		–	–
Yes	1.08 (0.93–1.27)	0.314	–	–
**YEAR OF DIAGNOSIS**				
2004–2006	1		1	
2007–2008	0.47 (0.37–0.59)	< 0.001	0.57 (0.44–0.75)	< 0.001
2009–2010	0.23 (0.17–0.30)	< 0.001	0.30 (0.22–0.42)	< 0.001
2011–2012	0.33 (0.26–0.41)	< 0.001	0.44 (0.34–0.57)	< 0.001
2013–2014	0.84 (0.72–0.99)	0.035	1.08 (0.88–1.33)	0.465

*ER, estrogen receptor*.

### Overall Survival

**Figure 2:**
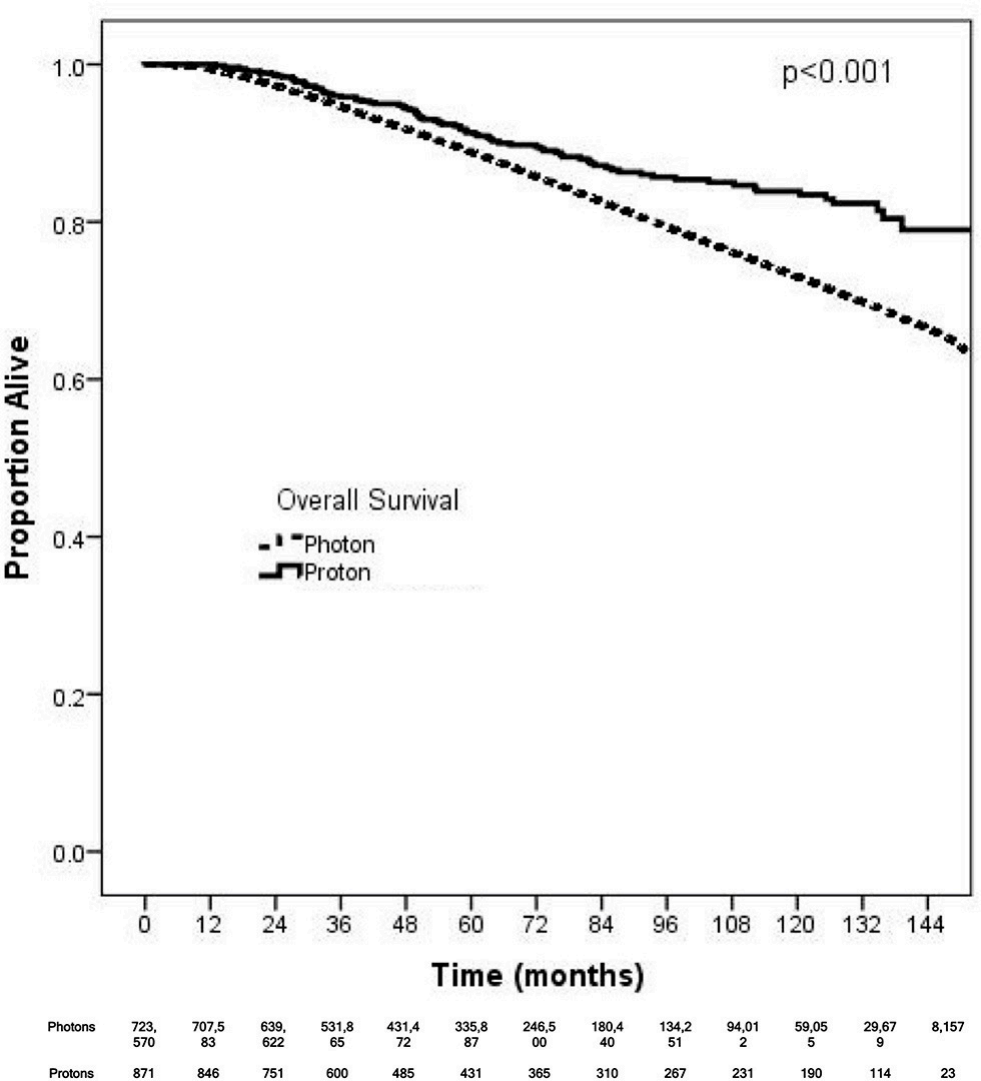
Kaplan-Meier survival analysis of breast cancer patients receiving adjuvant proton and non-proton radiotherapy.

On unadjusted, Kaplan Meier analysis, 5-year OS with PRT and EBRT were 91.9 and 88.9%, respectively (*p* < 0.001; Figure 2). On univariate analysis for OS, all other variables included were also significant predictors. MVA also demonstrated nearly all factors as significant predictors for OS (Table 2), except for percentage with no high school education (*p* = 0.477) and laterality (*p* = 0.070). PRT, however, was not a significant predictor for OS on MVA: hazard ratio (HR) = 0.85, 95% confidence interval (CI): 0.68–1.07, *p* = 0.168.

**Table 2:** Multivariable Analysis for overall survival in non-metastatic breast cancer patients treated with adjuvant radiation therapy.

	**Hazard ratio (95% Confidence interval)**	**P-value**
**TYPE OF RADIATION THERAPY**		
Photons	1	
Protons	0.85 (0.68–1.07)	0.168
**AGE**		
≤ 65	1	
>65	1.90 (1.86–1.94)	< 0.001
**RACE**		
White	1	
Black	1.16 (1.14–1.19)	< 0.001
Other	0.77 (0.74–0.80)	< 0.001
**INSURANCE STATUS**		
None	1	
Private	0.69 (0.66–0.73)	< 0.001
Medicaid	1.05 (0.99–1.11)	0.111
Medicare	1.01 (0.96–1.07)	0.621
Other	0.82 (0.77–0.88)	< 0.001
**CHARLSON-DEYO COMORBIDITY SCORE**		
0	1	
1	1.40 (1.37–1.43)	< 0.001
≥2	2.12 (2.05–2.20)	< 0.001
**FACILITY**		
Non-academic	1	
Academic	0.87 (0.86–0.89)	< 0.001
**MEDIAN HOUSEHOLD INCOME**		
< $38,000	1	
$38,000–$47,999	0.98 (0.96–1.01)	0.151
$48,000–$62,999	0.92 (0.90–0.94)	< 0.001
≥$63,000	0.80 (0.78–0.82)	< 0.001
**LOCATION**		
Northeast	1	
Midwest	1.06 (1.04–1.09)	< 0.001
South	1.08 (1.06–1.10)	< 0.001
West	0.88 (0.86–0.91)	< 0.001
**RESIDENCE**		
Metropolitan	1	
Urban	0.96 (0.94–0.98)	< 0.001
Rural	0.87 (0.83–0.92)	< 0.001
**PERCENTAGE WITH NO HIGH SCHOOL EDUCATION**		
≥13%	1	
< 13%	0.99 (0.98–1.01)	0.477
**LATERALITY**		
Right	1	
Left	1.01 (1.00–1.03)	0.070
**pT-STAGE**		
pT0	1	
pTis	0.70 (0.60–0.80)	< 0.001
pT1	0.67 (0.61–0.74)	< 0.001
pT2	0.90 (0.82–1.00)	0.039
pT3	1.12 (1.02–1.24)	0.022
pT4	1.52 (1.37–1.68)	< 0.001
**pN-STAGE**		
pN0	1	
pN1	1.33 (1.30–1.37)	< 0.001
pN2	1.84 (1.75–1.92)	< 0.001
pN3	2.69 (2.57–2.82)	< 0.001
**OVERALL STAGE**		
0	1		
I	1.73 (1.50–2.00)	< 0.001
II	2.20 (1.91–2.53)	< 0.001
III	2.37 (2.03–2.75)	< 0.001
**RECEPTOR STATUS**		
ER–	1	
ER+	0.69 (0.67–0.70)	< 0.001
Borderline/Unknown	0.74 (0.72–0.76)	< 0.001
**CHEMOTHERAPY**		
No	1	
Yes	0.69 (0.67–0.70)	< 0.001
**ENDOCRINE THERAPY**		
No	1	
Yes	0.64 (0.63–0.66)	< 0.001
**SURGERY**		
Breast Conservation	1	
Mastectomy	1.35 (1.32–1.38)	< 0.001
**YEAR OF DIAGNOSIS**		
2004–2006	1	
2007–2008	1.06 (1.04–1.08)	< 0.001
2009–2010	0.86 (0.84–0.88)	< 0.001
2011–2012	0.74 (0.72–0.76)	< 0.001
2013–2014	0.74 (0.71–0.77)	< 0.001

*ER, estrogen receptor*.

Kaplan Meier analyses were also performed after stratifying for factors associated with increase heart doses. On unadjusted analyses, PRT was associated with a higher survival (all *p* < 0.05) for patients with left-sided tumors, right-sided tumors, inner quadrant tumors, and breast conservation. PRT was not significant for improved OS for patients undergoing mastectomy (*p* = 0.067), pathologic node positive (*p* = 0.169), pathologic N2-N3 disease (*p* = 0.131), and regional lymph node irradiation (LNI) (*p* = 0.143). Supplemental Figures 1–5 illustrates the OS of PRT and EBRT for each of these subsets. Table 3 demonstrates MVA for OS for all patients and after including stratification criteria. On MVA, PRT no longer remained significant for OS within any of the stratified subsets.

**Table 3:** Multi-variate analysis examining the effect of protons, relative to non-protons, on overall survival.

**Variable**	**Stratification criteria**	**Hazard ratio for OS with protons (relative to non-protons)**	**95% Confidence interval**	***p*-value**
Laterality	Left sided	0.78	0.57–1.08	0.140
	Right sided	0.93	0.68–1.28	0.671
Quadrant location	Inner	0.60	0.28–1.25	0.173
	Outer	0.48	0.15–1.48	0.199
Type of surgery	Mastectomy	0.79	0.60–1.04	0.095
	Breast conservation	1.03	0.69–1.54	0.886
Nodal status	Node positive	1.07	0.77–1.50	0.680
	Node Negative	0.75	0.55–1.02	0.066
N2-N3 status	Positive	1.04	0.65–1.65	0.880
	Negative	0.81	0.63–1.05	0.118
Type of radiation	Breast and Lymph nodes	0.94	0.61–1.44	0.767
	Breast only	0.82	0.63–1.07	0.143

*Patients are also stratified into subgroups that are likely at risk for higher heart doses. The first listed variable is the subgroup at more risk*.

## Discussion

PRT is an exciting therapeutic option due to its ability to minimize exit dose and thereby reduce radiation dose to critical organs. Specifically for breast cancer patients, PRT may decrease dose to the lungs and heart, which can potentially decrease morbidity and possibly mortality (20). At this time, proton therapy is not accessible to all patients as only 27 proton therapy centers are currently operational in the United States. As such, the aim of this study was to investigate utilization patterns of protons and compare OS between PRT and EBRT. This present study is the largest to examine PRT for breast cancer to date, identifying 871 patients. Over half of the PRT patients (58.3%) were stage 0–1; early T stage also predicted for PRT. PRT was not associated with improved OS after adjusting for other factors on MVA. While some factors associated with higher heart doses—mastectomy and left sided tumors—also predicted for PRT, these subsets did not appear to have an OS benefit with protons on MVA.

Patients who are most likely benefit from PRT are those expected to have high heart doses. Numerous studies suggest that factors associated with relatively increased heart doses have been identified: left sided tumors, inner quadrant tumors, undergoing mastectomy, and receipt of regional nodal irradiation (21). This is consistent with our finding that predictors for receipt of PRT include patients with left sided tumors and undergoing mastectomy. The factor associated with the highest dose of radiation to the heart—LNI, particularly internal mammary lymph node (IMN) radiation (21)—was not a predictive of receipt of therapy in our analysis. One possible explanation is the NCDB does not specify the exact lymph node basin receiving therapy, but rather if the regional lymph nodes were irradiated or not. Alternatively, the median follow up−62.2 months may not be long enough to develop cardiac toxicities that affect OS (22). Contrary to this, Darby et al. dedicated analysis and follow up for major cardiac toxicity demonstrated that they begin within 5 years of RT (2).

In this analysis, PRT was not associated with improved OS on MVA for all breast cancer patients. Our population included patients with overall stage 0–3 breast cancer. Patients with early stage breast cancer receiving RT to the intact breast alone have been shown to have minimal heart doses. Indeed, a study by Kozak et al. demonstrated that while PRT did decrease heart doses for early stage patients, the mean heart doses and absolute reduction were minimal (mean heart dose with protons vs. photons: 0.1 vs. 0.4 Gy, reduction of 0.3 Gy) (7). In light of this small dosimetric benefit for early stage breast, the finding that 58.3% (508 out of 871) of the PRT cohort was stage 0–1 is provocative because these patients may have minimal clinical benefit; furthermore, our analysis for predictors of type of RT did not demonstrate that lower group stages of breast cancer inversely predicted for less likely utilization of protons.

Since the Kozak publication, studies have appeared to focus on clinical scenarios associated with higher mean heart doses, particularly treatment of the chest wall after mastectomy and treatment of the IMN lymph nodes. In the post-mastectomy setting, protons resulted in a greater reduction in mean heart dose: 2.0 vs. 0.2 Gy (8). Bradley et al also demonstrated that protons are associated with lower mean heart doses relative to photon-based RT: (0.5 vs. 2.9 Gy for right sided tumors; 0.6 vs. 5.9 Gy for left sided tumors) (6). Building on the Bradley study, Stick et al. (10) performed a comparative analysis of proton with photons for IMN and combined this with the Darby et al. (2) predictive model for risk of cardiac toxicity from RT. They demonstrated that PRT for IMN reduced the risk of toxicity by 2.9% relative to photons and improved breast cancer recurrence risk by 0.9%. Together, these studies suggest protons may improve the therapeutic benefit/risk ratio in the post-mastectomy and IMN radiation settings. To test this hypothesis, we performed a subset analysis. 203 proton patients had a mastectomy (23.3%), while 206 received LNI therapy (23.7%) (Supplemental Table 1). In both of these subsets, PRT, however, was not a predictor for improved OS on adjusted, MVA (Table 3). Furthermore, in another potential subset of patients likely to receive IMN radiation—N2-N3 patients—PRT remained not associated with OS. These negative findings suggest PRT may not translate into an OS benefit even for patients at risk of higher heart doses. Alternatively, any possible benefit with PRT may require a large sample size: although this is the largest study of PRT for breast cancer (*n* = 871) to date, only a percentage of these were likely patients at risk for higher heart doses. With PRT machine costing up to 40x more than a traditional photon linear accelerator, it is unclear, however, if the number needed to treat is worth the cost.

Mailhot Vega et al examined the cost effectiveness of PRT for breast cancer. For patients with a mean heart dose less than 5 Gy, PRT was not cost effective (23). Of the subsets at increased risk of elevated heart doses—left sided, post-mastectomy, inner quadrant and IMN radiation—only IMN irradiation is consistently associated with mean heart doses greater than 5 Gy (21). Patients who are recommended IMN RT and with an anticipated heart dose >5 Gy may economically benefit from protons, albeit at a cost of $100,000/life year. Furthermore, the advantage of IMN radiation is unclear: the Danish population study (showing an OS benefit) (4), and French randomized study (showing no OS benefit) (24), both of which included patients who underwent minimum chest wall + axillary and supraclavicular ± IMN irradiation, offered conflicting conclusions. In light of this unclear benefit of IMN therapy and protons' cost and clinical benefit predominantly in the setting of IMN irradiation, our findings of no OS advantage associated with protons further provides caution of using PRT for well-selected breast cancer outside of randomized clinical trials.

Our study has several limitations. The ability of PRT to lower toxicity is due to lower exit dose to the heart and lung. A major limitation of our study is that the NCDB does not capture this important toxicity data. Our follow-up time is 62.2 months, which is potentially too short to capture cardiac events. In addition, there is a likely an inherent selection bias regarding treatment allocation. However, this bias did not translate into an OS benefit on MVA. We could not account for unmeasured cofounders, including functional status, facility volume, clinician expertise, or availability of technology. Technology is also an important unaccounted factor in this analysis: deep inspiratory breath hold is a technique commonly used that lowers dose to the heart and lungs (3). The recent study by Liao et al also suggests that experience and to a lesser extent facility volume improve delivery of PRT (25). Improved delivery of PRT may also affects heart and lung doses in breast cancer patients. Her2 receptor status is not coded in the NCDB and may be a relevant factor, especially since Her2 targeted therapy can increase heart toxicity (26). These factors are all potential confounding variables that limit the OS analysis of this study.

## Conclusions

In conclusion, protons were associated with left sided tumor and receipt of mastectomy, but not higher group stages or LNI. However, PRT for all breast cancer patients and cohorts at risk for higher heart doses was not a significant predictor for improved OS on MVA. In light of the high cost of proton RT, these data question the utilization of PRT, especially in early stage patients with expected low heart doses. Given the limitations of the NCDB, it is still possible that the dosimetric benefit of PRT translates into a clinical benefit for patients besides for OS, including toxicity and/or local control, particularly in those with anticipated higher heart doses. Therefore, PRT should remain to be an active area of research. We support enrollment on RTOG 3510, which compares PRT vs. photons in patients receiving IMN RT (20); this study will help determine if PRT helps decrease heart toxicity in the breast cancer patient population at highest risk for radiation related major cardiac events and if this leads to an OS benefit.

## Author Contributions

MC, AL, and KP: study design, data interpretation, manuscript development, literature review, tables and figures, final manuscript. SG, DW, PB, RD, NB, HP, JY, LW, MM, SH, and CK: data interpretation, manuscript development, final manuscript.

### Conflict of Interest Statement

The authors declare that the research was conducted in the absence of any commercial or financial relationships that could be construed as a potential conflict of interest.
