# Supplementary material for: Is Proton Therapy a “Pro” for Breast Cancer? A Comparison of Proton vs. Non-proton Radiotherapy Using the National Cancer Database
Source: Front Oncol. 2019 Jan 14;8:678. doi: 10.3389/fonc.2018.00678 (PMC6339938; doi:10.3389/fonc.2018.00678)
Supplement: Supplementary file 1 [file Table_1.DOCX]

| Characteristic | Received Photon Therapy (n=723,621) | Received Proton Therapy (n=871) | p-value |
| --- | --- | --- | --- |
| Median age (years) | 60 | 59 | 0.074 |
| Race |  |  | <0.001 |
| White | 606,173  (83.8%) | 737  (84.6%) |  |
| Black | 80,265  (11.1%) | 56  (6.4%) |  |
| Other | 37,183  (5.1%) | 78  (9.0%) |  |
| Insurance status |  |  | 0.038 |
| None | 13,765  (1.9%) | 11  (1.3%) |  |
| Private | 414,234  (57.2%) | 542  (62.2%) |  |
| Medicaid | 44,170  (6.1%) | 49  (5.6%) |  |
| Medicare | 234,719  (32.4%) | 254  (29.2%) |  |
| Other/unknown | 16,733  (2.3%) | 15  (1.7%) |  |
| Charlson-Deyo comorbidity score |  |  | <0.001 |
| 0 | 624,930  (86.4%) | 790  (90.7%) |  |
| 1 | 82,957  (11.5%) | 61  (7.0%) |  |
| >=2 | 15,734  (2.2%) | 20  (2.3%) |  |
| Facility |  |  | <0.001 |
| Non-academic | 481095  (66.5%) | 465  (53.4%) |  |
| Academic | 209422  (28.9%) | 353  (40.5%) |  |
| Unknown | 33104  (4.6%) | 53  (6.1%) |  |
| Median household income |  |  | <0.001 |
| <$38,000 | 100779  (13.9%) | 85  (9.8%) |  |
| $38,000-$47,999 | 148077  (20.5%) | 132  (15.2%) |  |
| $48,000-$62,999 | 192511  (26.6%) | 157  (18.0%) |  |
| >=$63,000 | 276127  (38.2%) | 490  (56.3%) |  |
| Unknown | 6127  (0.8%) | 7  (0.8%) |  |
| Location |  |  | <0.001 |
| Northeast | 167253  (23.1%) | 74  (8.5%) |  |
| Midwest | 184124  (25.4%) | 86  (9.9%) |  |
| South | 221128  (30.6%) | 147  (16.9%) |  |
| West | 118012  (16.3%) | 511  (58.7%) |  |
| Unknown | 33104  (4.6%) | 53  (6.1%) |  |
| Residence |  |  | <0.001 |
| Metropolitan | 608,387  (86.7%) | 806  (94.6%) |  |
| Urban | 83,608  (11.9%) | 39  (4.6%) |  |
| Rural | 10,075  (1.4%) | 7  (0.8%) |  |
| Percentage with no high school education |  |  | 0.137 |
| >=13% | 263034  (36.3%) | 338  (38.8%) |  |
| <13% | 454737  (62.8%) | 526  (60.4%) |  |
| Unknown | 5850  (0.8%) | 7  (0.8%) |  |
| Laterality |  |  | 0.036 |
| Right | 358,214  (49.5%) | 400  (45.9%) |  |
| Left | 365,407  (50.5%) | 471  (54.1%) |  |
| pT-stage |  |  | 0.001 |
| pT0 | 13,623  (1.9%) | 25  (2.9%) |  |
| pTis | 72,551  (10.0%) | 117  (13.4%) |  |
| pT1 | 424,195  (58.6%) | 495  (56.8%) |  |
| pT2 | 169,160  (23.4%) | 180  (20.7%) |  |
| pT3 | 33,119  (4.6%) | 46  (5.3%) |  |
| pT4 | 10,973  (1.5%) | 8  (0.9%) |  |
| pN-stage |  |  | 0.305 |
| pN0 | 498,945  (69.0%) | 608  (69.8%) |  |
| pN1 | 144,685  (20.0%) | 155  (17.8%) |  |
| pN2 | 53,918  (7.5%) | 73  (8.4%) |  |
| pN3 | 26,073  (3.6%) | 35  (4.0%) |  |
| Overall stage |  |  | <0.001 |
| 0 | 73072  (10.1%) | 118  (13.5%) |  |
| I | 334,545  (46.2%) | 390  (44.8%) |  |
| II | 197,693  (27.3%) | 200  (23.0%) |  |
| III | 92,390  (12.8%) | 132  (15.2%) |  |
| Unknown | 25,921  (3.6%) | 31  (3.6%) |  |
| Receptor status |  |  | <0.001 |
| ER- | 119,628 (16.5%) | 96 (11.0%) |  |
| ER+ | 535,875 (74.1%) | 586 (67.3%) |  |
| Borderline/ | 68,118 (9.4%) | 189 (21.7%) |  |
| Unknown |  |  |  |
| Chemotherapy |  |  | 0.093 |
| No | 379284 (52.4%) | 482 (55.3%) |  |
| Yes | 330471 (45.7%) | 374 (42.9%) |  |
| Unknown | 13866 (1.9%) | 15 (1.7%) |  |
| Endocrine therapy |  |  | <0.001 |
| No | 201961 (27.9%) | 300 (34.4%) |  |
| Yes | 498802 (68.9%) | 557 (63.9%) |  |
| Unknown | 22858 (3.2%) | 14 (1.6%) |  |
| Surgery |  |  | 0.017 |
| Breast-conserving surgery | 578222 (79.9%) | 667 (76.6%) |  |
| Mastectomy | 144944 (20.0%) | 203 (23.3%) |  |
| Not Specified | 455 (0.1%) | 1 (0.1%) |  |
| Lymph Node Irradiation |  |  | 0.308 |
| No | 562,762 (77.8%) | 665 (76.3%) |  |
| Yes | 160,859 (22.2%) | 206 (23.7%) |  |
| Year of diagnosis |  |  | <0.001 |
| 2004-2006 | 157,890 (21.8%) | 316 (36.3%) |  |
| 2007-2008 | 97,371 (13.5%) | 91 (10.4%) |  |
| 2009-2010 | 126,769 (17.5%) | 58 (6.7%) |  |
| 2011-2012 | 164,451 (22.7%) | 107 (12.3%) |  |
| 2013-2014 | 177,140 (24.5%) | 299 (34.3%) |  |

**Supplemental Table 1** – Comparing baseline characteristics of breast cancer patients treated with post-operative proton or non-proton radiation therapy.

ER = estrogen receptor.
