# Supplementary material for: Is Proton Therapy a “Pro” for Breast Cancer? A Comparison of Proton vs. Non-proton Radiotherapy Using the National Cancer Database
Source: Front Oncol. 2019 Jan 14;8:678. doi: 10.3389/fonc.2018.00678 (PMC6339938; doi:10.3389/fonc.2018.00678)
Supplement: Supplementary file 2 [file Image_1.pdf]

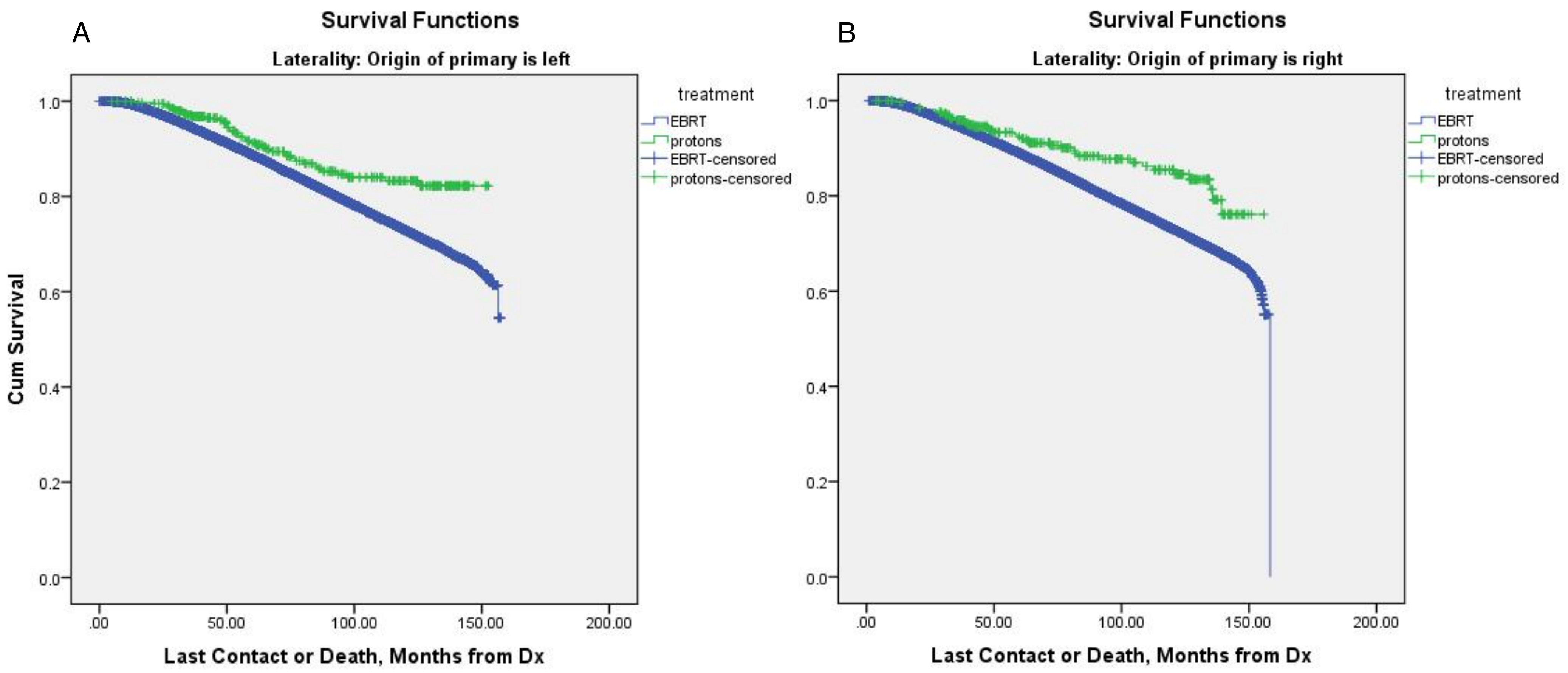

Supplemental Figure 1: Overall Survival for Left (A) vs. Right (B) Sided Tumors with Proton vs. Non-Proton EBRT

Left-sided Cohort

5-year Overall Survival

- Non-Proton (EBRT): 88.7%
- Protons: 91.7%

p-value=0.001

Right Sided Cohort

5-year Overall Survival

- Non-Proton (EBRT): 89.0%
- Protons: 92.5%

p-value=0.001
