# Supplementary material for: Is Proton Therapy a “Pro” for Breast Cancer? A Comparison of Proton vs. Non-proton Radiotherapy Using the National Cancer Database
Source: Front Oncol. 2019 Jan 14;8:678. doi: 10.3389/fonc.2018.00678 (PMC6339938; doi:10.3389/fonc.2018.00678)
Supplement: Supplementary file 3 [file Image_2.pdf]

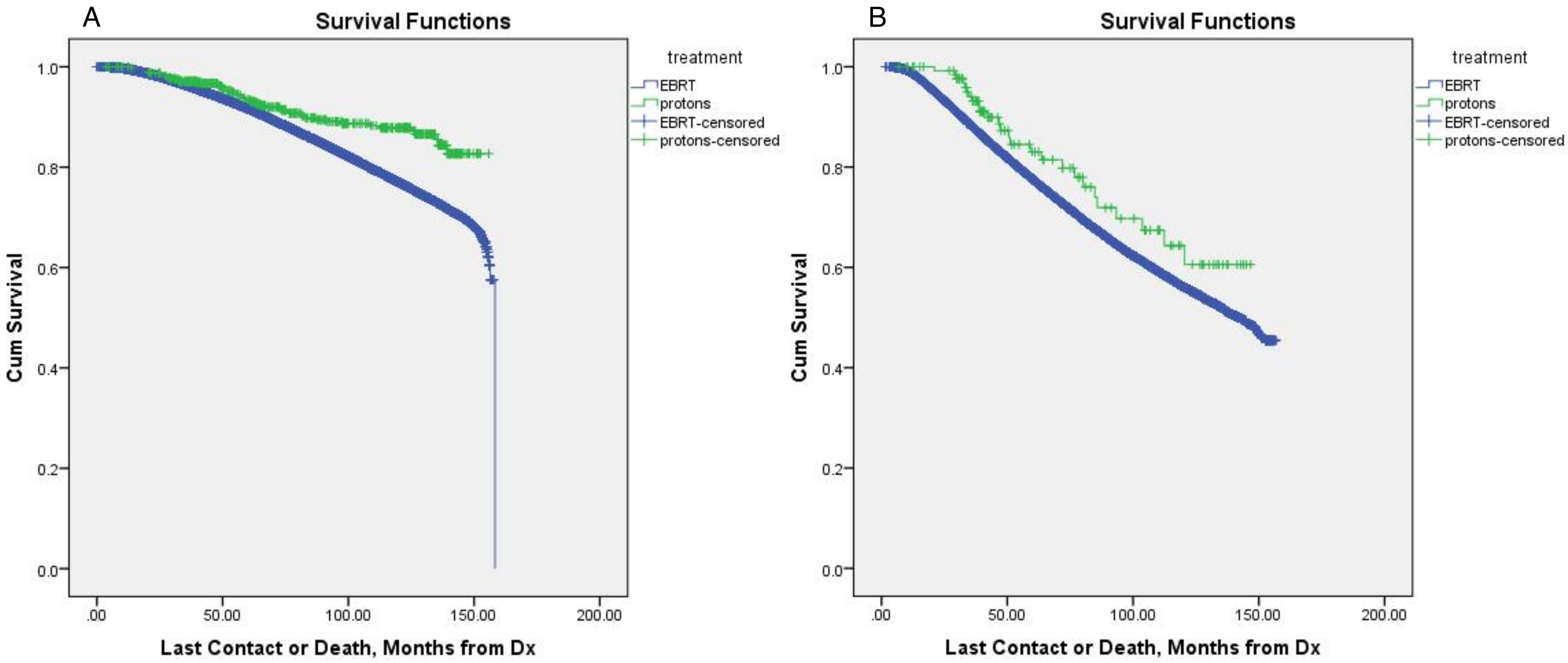

Supplemental Figure 2: Overall Survival with Proton vs. Non-Proton EBRT in Breast Conversation (A) and Mastectomy (B) Cohorts

Breast Conservation Cohort

5-year Overall Survival

- Non-Proton (EBRT): 91.7%
- Protons: 92.3%

p-value<0.001

Mastectomy Cohort

5-year Overall Survival

- Non-Proton (EBRT): 77.6%
- Protons: 83.0%

p-value=0.067
