# Supplementary material for: Is Proton Therapy a “Pro” for Breast Cancer? A Comparison of Proton vs. Non-proton Radiotherapy Using the National Cancer Database
Source: Front Oncol. 2019 Jan 14;8:678. doi: 10.3389/fonc.2018.00678 (PMC6339938; doi:10.3389/fonc.2018.00678)
Supplement: Supplementary file 4 [file Image_3.pdf]

A

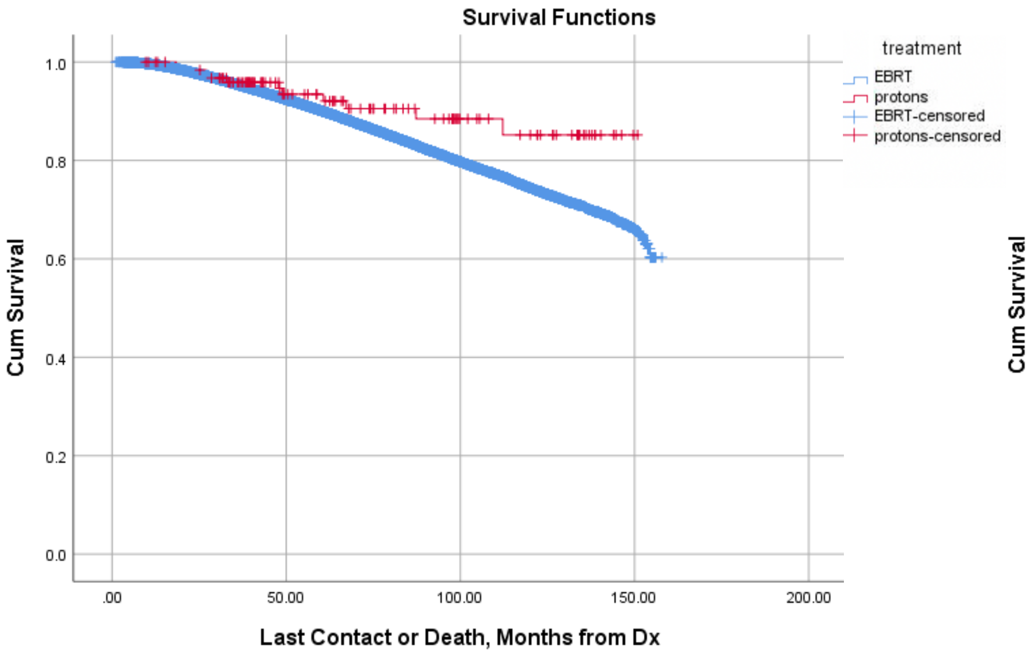

B

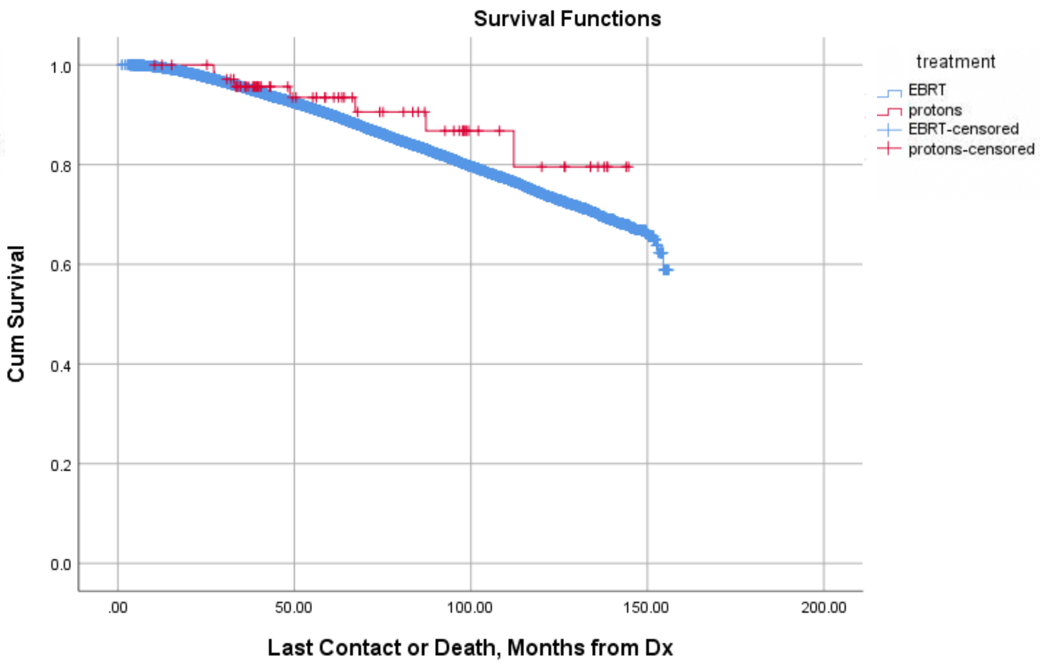

Supplemental Figure 3: Overall Survival with Proton vs. Non-Proton EBRT in Tumors located in Inner Quadrant (A) and Outer Quadrant (B) Cohorts

Inner Quadrant Cohort

5-year Overall Survival

- Non-Proton (EBRT): 90.1%
- Protons: 93.5%

p-value=0.055

Outer Quadrant Cohort

5-year Overall Survival

- Non-Proton (EBRT): 91.6%
- Protons: 93.2%

p-value=0.276
