# Supplementary material for: Is Proton Therapy a “Pro” for Breast Cancer? A Comparison of Proton vs. Non-proton Radiotherapy Using the National Cancer Database
Source: Front Oncol. 2019 Jan 14;8:678. doi: 10.3389/fonc.2018.00678 (PMC6339938; doi:10.3389/fonc.2018.00678)
Supplement: Supplementary file 5 [file Image_4.pdf]

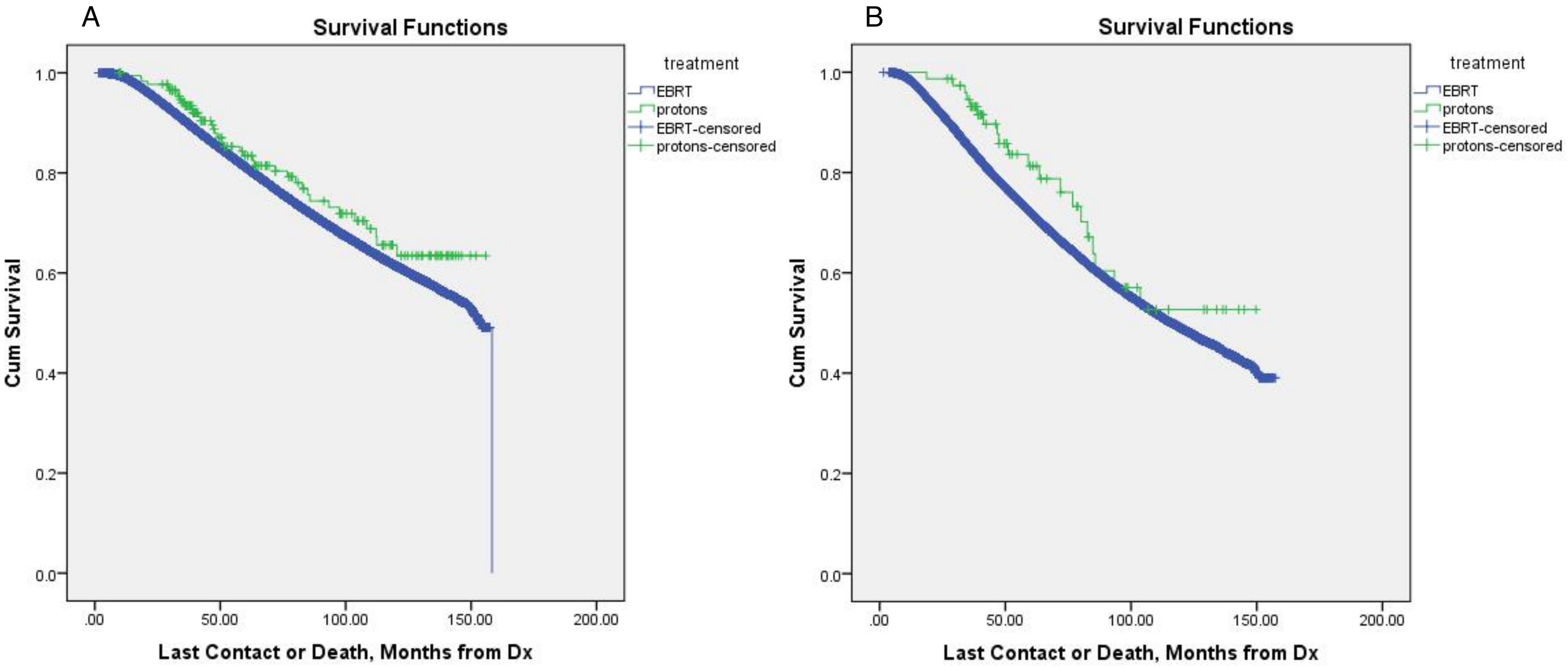

Supplemental Figure 4: Overall Survival with Proton vs. Non-Proton EBRT in Pathologically Node Positive (A) and Pathologically N2-N3 Disease (B) Cohorts

Pathologically Node Positive Cohort

5-year Overall Survival
- Non-Proton (EBRT): 81.2%
- Protons: 83.4%

p-value=0.169

Pathologically N2-N3 disease Cohort

5-year Overall Survival
- Non-Proton (EBRT): 72.0%
- Protons 81.3%

p-value=0.131
