# Supplementary material for: Is Proton Therapy a “Pro” for Breast Cancer? A Comparison of Proton vs. Non-proton Radiotherapy Using the National Cancer Database
Source: Front Oncol. 2019 Jan 14;8:678. doi: 10.3389/fonc.2018.00678 (PMC6339938; doi:10.3389/fonc.2018.00678)
Supplement: Supplementary file 6 [file Image_5.pdf]

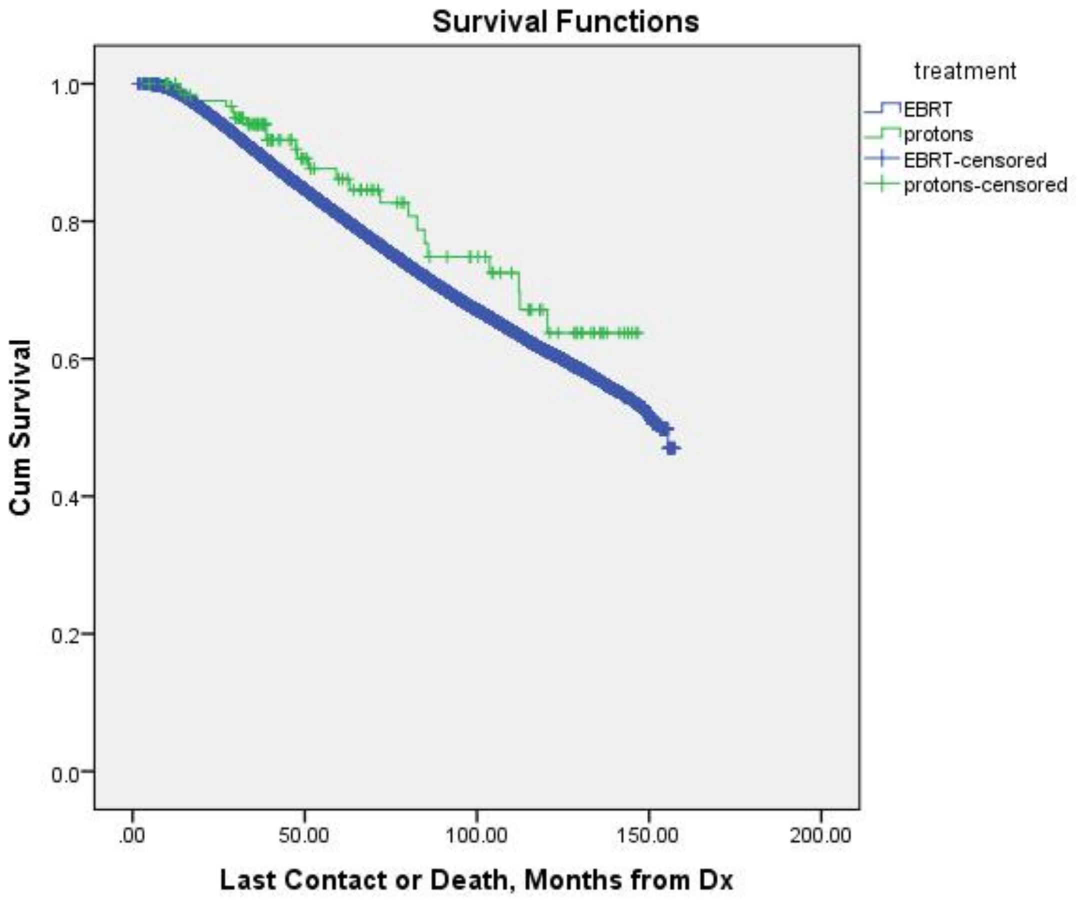

Supplemental Figure 5: Overall Survival with Proton vs. Non-Proton EBRT in Received Lymph Node Irradiation Cohort

5-year Overall Survival

- Non-Proton (EBRT): 80.8%
- Protons: 86.1%

p-value=0.140
